# Role of thwarted belongingness, perceived burdensomeness and psychological distress in the association between adverse childhood experiences and suicidal ideation in college students

**DOI:** 10.1192/bjo.2021.1087

**Published:** 2022-02-03

**Authors:** Madhav Bhargav, Lorraine Swords

**Affiliations:** School of Psychology, Trinity College Dublin, University of Dublin, Ireland; School of Psychology, Trinity College Dublin, University of Dublin, Ireland; and Trinity Research in Childhood Centre, Trinity College Dublin, University of Dublin, Ireland

**Keywords:** Adverse childhood experiences, college students, mental health, perceived burdensomeness, suicide.

## Abstract

**Background:**

Adverse childhood experiences (ACEs) have a detrimental impact on short- and long-term mental and physical health. A growing body of research has indicated that the prevalence of suicidal phenomena is significantly higher among individuals with a history of ACEs. However, there is a lack of understanding about processes that result in ACEs leading to suicidal ideation when testing within a theoretical framework.

**Aims:**

To develop and test a multidimensional model that would explain the association between ACEs and suicidal ideation in college students.

**Method:**

Data were obtained from a cross-sectional survey completed by 321 college students primarily recruited from universities in Ireland. Participants were aged 18–21 (*n* = 176) and 22–25 years (*n* = 145). An ACEs questionnaire, the Interpersonal Needs Questionnaire, which assessed thwarted belongingness and perceived burdensomeness, the CORE-10, which assessed psychological distress, and the Suicide Ideation Scale (SIS) were administered.

**Results:**

After controlling for gender and sexual orientation, results revealed a significant direct effect of ACEs on suicidal ideation such that more accumulated ACEs were associated with higher suicidal ideation (effect size 0.30; 95% CI 0.047–0.538). A significant indirect effect of ACEs on suicidal ideation through perceived burdensomeness and psychological distress, and thwarted belongingness and psychological distress, was observed (effect size 0.90; 95% CI 0.558–1.270).

**Conclusions:**

Findings suggest that ACEs have a detrimental impact on college students’ mental health. Results highlight the potential benefits of ACE-informed interventions that target thwarted belongingness and perceived burdensomeness to countervail suicidal ideation in college students.

Suicide has become one of the leading causes of death across all ages worldwide. Among those aged between 15 and 29 years, suicide is ranked second as a cause of death globally.^1^ Suicidal ideation, which is defined as thoughts to end one's own life, is a pivotal precursor to later attempted and completed suicide, and is therefore considered to be a major public health matter.^2^ Among college students it is estimated that 15–30% have reported some degree of suicidal thoughts.^3^

## The interpersonal theory of suicide

There is growing empirical support for the interpersonal theory of suicide^4^ in explaining suicidality among college students.^5^ This theory proposes that two cognitive-affective states are key for an individual to think about, attempt or die by suicide.^6^ These two interpersonally aligned factors are perceived burdensomeness, where the person believes themselves to be a burden on others, and thwarted belongingness, where the person perceives a disconnection in their closeness with others or a lack of reciprocity in caring. To identify and mitigate suicide risk among college students, it is important to examine the extent of their suicidal ideation and understand the factors and processes that may place some students at heightened risk.

## College students’ mental health

Success or failure in college can make a substantial difference in individuals’ developmental trajectories.^7^ Although higher education is associated with personal and professional advantages, the college years for some students are characterised by a range of stressors, such as economic challenges, excessive alcohol use, social isolation and psychological distress.^8^ Students who have accumulated a number of negative and potentially traumatic life events may be particularly vulnerable to the effects of such stresses^9^ and be at heightened suicide risk.^10^ In this regard, more than 95% of counselling centre directors in a US survey reported that meeting the needs of university students with significant psychological problems is a growing concern on campus.^11^ A longitudinal study (*n* = 155 206) revealed an increase across all measures of service utilisation among college students, from 19% in 2007 to 34% in 2017, with similar patterns for both therapy/counselling and medication use.^12^

## Adverse childhood experiences and later mental health

Adverse childhood experiences (ACEs) refer to negative life events that individuals encounter during their first 18 years of life.^13^ They include neglect or maltreatment and significant dysfunction in the home, such as domestic violence, drug or alcohol addiction and parental incarceration. ACEs tend to co-occur and a significant body of research has demonstrated their cumulative effect, where each additional adversity experienced in childhood increases the risk of poorer physical, psychological and behavioural outcomes into and throughout adulthood.^13,14^

A growing body of research has established a relationship between ACEs and suicidal thoughts and behaviours and has indicated that the prevalence of suicidal phenomena is significantly higher among individuals with a history of ACEs.^15^ One key study, which included 9377 participants from the UK 1958 British Birth Cohort, prospectively assessed childhood adversity at 7, 11 and 16 years of age and suicidal ideation at ‘mid-life’ (45 years of age) and noted how accumulating three or more childhood adversities was associated with greater later suicidal ideation.^16^ The study also established that other factors, such as internalising and externalising disorders and interpersonal cognitive-affective states, partially mediated this relationship.

Young people with multiple accumulated ACEs, or different constellations of ACEs, may find the transition to college and the more stressful aspects of college life particularly challenging.^17^ Some studies have shown high rates of ACEs in the college-going population, for example between 35 and 40% of students reporting that they had experienced two or more ACEs.^18^ The relationship between ACEs and poorer mental health and suicidality among students has also been established.^19^

## The present study

To date, there has been a lack of understanding with regard to differentiating and identifying the suicidal phenomena of suicidal ideation and suicide attempt and testing their relationship with cumulative exposure to ACEs. This distinction is pivotal for both theoretical and practical reasons. The present study focuses on suicidal ideation. ‘Ideation-to-action’ frameworks of suicide^20^ propose that suicidal thinking is one of the first steps on the pathway to suicidal behaviours.

The central aims of this study are to (a) report on the extent of ACEs, psychological distress and suicidal ideation in a college-going population, (b) examine the role of psychological distress in mediating the relationship between ACEs and suicidal ideation and (c) test a serial mediation model examining the indirect effects of thwarted belongingness, perceived burdensomeness and psychological distress to determine whether thwarted belongingness and perceived burdensomeness mediated the relationship between ACEs and suicidal ideation via psychological distress.

**Fig. 1:**
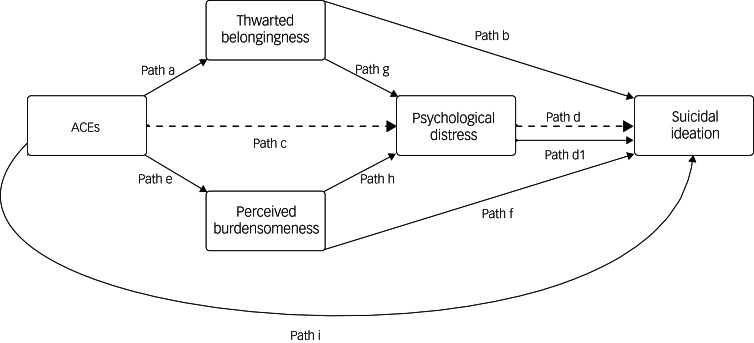
Conceptual diagram of Hypothesis 1 (dotted lines) and 2 (bold lines). ACEs, adverse childhood experiences.

Based on the previous literature associated with ACEs, suicidal ideation and psychological distress, we first hypothesised was that there would be a significant, positive and unique indirect effect of psychological distress in the relationship between cumulative exposure to ACEs and suicidal ideation in college students (Fig. 1, Path c → Path d). Our second hypothesis was based on the interpersonal theory of suicide model stating that there would be a significant, positive indirect effect of thwarted belongingness in serial, and a significant, positive indirect effect of perceived burdensomeness in serial, in the relationship between cumulative exposure to ACEs and suicidal ideation via psychological distress in college students (Fig. 1, Path a → Path g → Path d_1_ and Path e → Path h → Path d_1_). These findings would suggest that thwarted belongingness, perceived burdensomeness and psychological distress explain the relationship between cumulative exposure to ACEs and current suicidal ideation.

## Method

### Participants

Participants were 321 college students primarily recruited from higher education institutions in Ireland. Their ages ranged from 18–21 years (*n* = 178) to 22–25 years (*n* = 145). Approximately two-thirds of participants identified as heterosexual (65.4%), while the remaining third (34.6%) identified as non-heterosexual, i.e. gay/lesbian, bisexual, asexual and others. The majority of participants identified themselves as females (78.8%); 18.1% identified themselves as males, and 3.1% identified as genderqueer, trans male, etc.

### Ethics

This study was performed in accordance with the Declaration of Helsinki and was approved by the School of Psychology research ethics committee at Trinity College Dublin (approval: SPREC092020-05). Data were collected through an online cross-sectional quantitative questionnaire and all participants took part voluntarily. Informed consent was obtained electronically after the participants had received a detailed introduction to the study.

### Measures

#### Demographics

Participants reported their age, sexual orientation, nationality, ethnicity and whether they were undergraduates or postgraduates. Data were also collected on each participant's living situation, any pre-existing mental or physical illnesses and monetary sufficiency.

#### Adverse childhood experiences

Cumulative exposure to ACEs was assessed using an adapted version of the ACEs Questionnaire,^13^ which assesses the presence or absence of maltreatment (e.g. emotional abuse, physical abuse, sexual abuse) and household dysfunction (e.g. substance misuse or domestic violence) before age 18. Questions are phrased such as ‘Did a parent or other adult in the household often or very often… swear at you, insult you, put you down or humiliate you?’ (emotional abuse item) or ‘act in a way that made you afraid that you might be physically hurt?’ (physical abuse item). Two additional questions were added to the original questionnaire in order to attain a more robust model when assessing the relationship between ACEs and psychological distress.^21^ These items were ‘Were your parents or step-parents arguing, yelling and angry at one another a lot of the time?’ and ‘Did your parents, brother or sister, or best friend suffer a “very bad illness” or “very bad accident” where they had to be in the hospital for a long time?’. The number of experiences reported by each participant was summed for a total ACEs score ranging from 0 to 12. ACEs questions have been used in several studies with young adults globally^19^ reporting acceptable reliability and validity.

#### Interpersonal needs

The Interpersonal Needs Questionnaire (INQ-15)^22^ is a 15-item scale comprising statements relating to perceived burdensomeness (e.g. ‘These days I think I am a burden on society’) and thwarted belongingness (e.g. ‘These days I feel disconnected from other people’). Participants respond on a 7-point ordinal response metric ranging from 1 (Not at all true of me) to 7 (Very true of me), with higher scores representing a greater feeling of perceived burdensomeness and thwarted belongingness. The INQ-15 subscales have consistently demonstrated good internal consistency, with Cronbach's alpha values ranging from 0.85 to 0.92 for perceived burdensomeness and 0.81 to 0.89 for thwarted belongingness.^23^ The Cronbach's alpha for the current sample was 0.95 for perceived burdensomeness and 0.88 for thwarted belongingness.

#### Psychological distress

The 10-item Clinical Outcomes in Routine Evaluation tool (CORE-10)^24^ assesses anxiety, depression, trauma, physical problems, general functioning and risk to self over the past week. Participants rate each item on a 5- point scale ranging from 0 (Not at all) to 4 (Most or all the time). For the final score, all items are added together to get the clinical score, with higher scores indicating higher distress, anxiety and depression. Statements include ‘I have felt I have someone to turn to for support when needed’ and ‘Talking to people has felt too much for me’. The CORE-10 internal reliability (alpha) is 0.90 and the score for the CORE-10 correlated with the CORE-OM (the longer scale that the CORE-10 is derived from) is 0.94 in a clinical sample and 0.92 in a non-clinical sample. The clinical cut-off score for general psychological distress has been measured as 11.0, with a reliable change index (90% CI) of 6. The Cronbach's alpha for the current sample was 0.85.

#### Suicidal Ideation Scale

The 10-item Suicidal Ideation Scale (SIS)^25^ measures suicidal ideation and behaviours. The SIS includes a 6-item subscale measuring resolved suicide preparations and behaviours (e.g. ‘I have made attempts to kill myself’) and a 4-item subscale measuring suicidal desire (e.g. ‘I feel life just isn't worth living’). Participants rate each item on a 5-point scale ranging from 1 (Never) to 5 (Always). Items are summed, with higher scores indicating a more severe risk for suicide. Total scores range from 10 to 50. Strong internal consistency (Cronbach's alpha of 0.86) and concurrent validity have been reported.^25^ Cronbach's alpha for the current sample was 0.89.

### Procedure

Participants were recruited via weekly emails sent by the College's Students’ Union and later via advertisement on social media. Students who gave web-based consent anonymously completed all questionnaires using a secure survey website called Qualtrics. Participants took between 8 and 12 min to complete the survey. At the end, participants were thanked for their time and directed to the debrief sheet, which included a list of mental health services and the researcher's contact details, should they have any follow-up questions about the research.

### Data analysis plan and data preparation

A serial mediation approach was used with PROCESS Model 80, v.3.5 in SPSS for MacOS^26^ that tested whether adversities in childhood predict suicidal ideation both directly and indirectly by way of thwarted belongingness, perceived burdensomeness and psychological distress individually, as well as by way of thwarted belongingness or perceived burdensomeness in combination with psychological distress. PROCESS does not require all variables to be normally distributed.^26^ To attain sample size requirements, PROCESS used bootstrapping to construct the 95% confidence interval at 5000 samples.

## Results

### ACEs, psychological distress and suicidal ideation

Childhood adversities were common, with students endorsing a mean of 2.94 ACEs and median of 2 ACEs, with 25.2% endorsing no ACE, 35.2% endorsing 1–3 ACEs and 39.6% endorsing 4–12 ACEs, out of a list of 12. The mean ACEs of those who reported at least 1 ACE was 3.94. Approximately 43% of the sample population reported some type of abuse (physical, sexual or emotional) and approximately 38% of the sample population reported some type of neglect (emotional or physical). Moreover, approximately 26.2% of respondents reported parental divorce and a similar proportion also reported alcohol misuse by household members.

The mean score of 18.25 on the CORE-10 (Table 1) indicates that on average students displayed moderate levels of psychological distress.^27^ Moreover, in relation to suicidal ideation, the current sample established a mean of 16.38. Based on results from the initial SIS validation study,^25^ scores greater than 1 s.d. above the mean (SIS total score of 15 or greater) are proposed to be indicative of serious suicidal ideation.^28^ See Table 1 for more descriptive information on the data.

**Table 1:** Descriptive statistics: achieved mean, s.d., and min–max of total score of the scales

Variables	*n*	Mean	s.d.	Minimum	Maximum
Adverse childhood experiences	321	2.94	2.62	0	9
Thwarted belongingness	321	31.30	12.04	9	63
Perceived burdensomeness	321	12.91	9.03	6	42
Psychological distress	321	18.25	7.90	1	39
Suicidal ideation	321	16.38	9.14	10	50

### Preliminary analysis

See Table 2 for partial bivariate correlations between all key study variables. As gender differences are frequently reported with regard to psychological distress and disorders in adolescents and young adults, participant gender (coded as female: 1; others: 0) was controlled for in the analyses. Sexual orientation (coded as heterosexual: 1; sexual minorities: 0) was also used as a covariate because it was significantly correlated with the outcome variable (suicidal ideation; *r* = –0.17, *P* = 002). This finding is consistent with previous literature that suggests that individuals from sexual minorities report a higher rate of thwarted belongingness, perceived burdensomeness and suicidal ideation when compared with those who identify themselves as heterosexual.^29^ Therefore, adjusting for gender and sexual orientation allowed for more accurate approximations of the relationship between childhood adversities and suicide ideation.

**Table 2:** Partial bivariate correlation for key study variables^a^

	1	2	3	4	5	6	7	8
1. Age	–							
2. Sexual orientation	0.012	–						
3. Gender	0.075	−0.096	–					
4. ACEs	0.160**	−0.064	−0.025	–				
5. INQ thwarted belongingness	0.039	−0.108	0.059	0.312**	–			
6. INQ perceived burdensomeness	0.113*	−0.111*	0.022	0.345**	0.617**	–		
7. CORE-10 psychological distress	−0.004	−0.157**	0.085	0.329**	0.588**	0.591**	–	
8. SIS suicidal ideation	0.132*	−0.114*	0.053	0.368**	0.493**	0.791**	0.584**	–

a. Age: coded as 18–21 years (1) and 22–25 years (0); sexual orientation: coded as heterosexual (1) and sexual minorities (0); gender: coded as female (1) and other (0); ACEs, Adverse Childhood Experiences questionnaire with two additional items; INQ, Interpersonal Needs Questionnaire score; CORE-10, 10-item Clinical Outcomes in Routine Evaluation tool score; SIS, Suicide Ideation Scale score.

** Correlation is significant at the 0.01 level.

* Correlation is significant at the 0.05 level.

### Mediation analysis

**Fig. 2:**
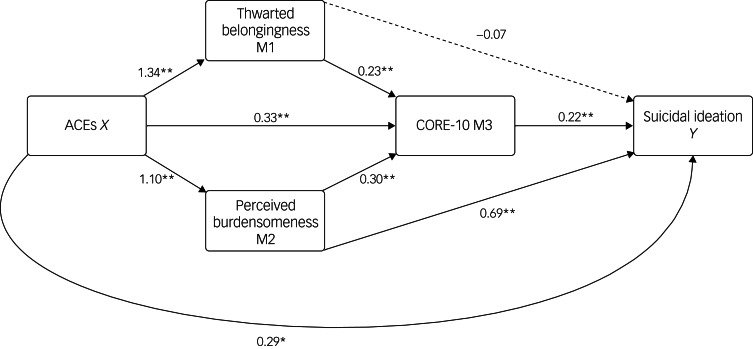
Unstandardized path coefficients of the mediating effects of thwarted belongingness, perceived burdensomeness, and psychological distress as measured by the psychological distress on the relationship between adversities in childhood and suicide ideation, after adjusting for gender (coded as females = 1 and others = 0) and sexual orientation (coded as heterosexuals = 1 and sexual minorities = 0). Non-significant paths are denoted with a dotted line. Total effect model summary: *F*(3, 317) = 21.84, *P* <.001, *R*^2^ = .17. ACEs, adverse childhood experiences.

The first regression was conducted on thwarted belongingness and ACEs (Fig. 2). ACE was a positive, significant predictor of thwarted belongingness (*b =* 1.34, *t*(317) = 5.47, *P* < 0.001). The second regression was conducted on perceived burdensomeness and ACEs, and ACE was a positive, significant predictor of perceived burdensomeness (*b =* 1.10, *t*(317) *=* 6.12, *P* < 0.001). The third regression was performed on CORE-10, which noted that: (a) ACE was a positive, significant predictor of CORE-10 score (*b =* 0.33, *t*(315) = 2.39, *P* =0.02); (b) thwarted belongingness was a positive, significant predictor of CORE-10 score (*b =* 0.23, *t*(315) = 6.39, *P* < 0.001); (c) perceived burdensomeness was a positive, significant predictor of CORE-10 score (*b =* 0.30, *t*(315) = 6.30, *P* < 0.001). These set of predictors accounted for 44.3% of variance in psychological distress *(F*(5, 315) = 50, *P* < 0.001). The final regression was performed on suicidal ideation, which noted that: (a) ACE was a positive, significant predictor of suicidal ideation (*b =* 0.29, *t*(314) = 2.34, *P* =0.02); (b) thwarted belongingness was not a significant predictor of suicidal ideation (*b* = −0.06, *t*(314) = −1.80, *P* =0.07); (c) perceived burdensomeness was a positive, significant predictor of suicidal ideation (*b* = 0.69, *t*(314) = 15, *P* < 0.001); (d) CORE-10 score was a positive, significant predictor of suicidal ideation (*b* = 0.22, *t*(314) = 4.38, *P* < 0.001). These set of predictors accounted for 66.01% of variance in suicidal ideation *(F*(6, 314) = 101.62, *P* < 0.001, *R*^2^ = 0.66.01).

### Indirect effects

This serial multiple mediator model consists of five indirect effects estimated as products of regression coefficients linking ACEs (*X*) to suicidal ideation (*Y*) (Table 3). The first indirect effect was interpreted as non-significant, meaning that thwarted belongingness did not mediate the relationship between ACEs and suicidal ideation (*X* → *M*_1_ → *Y*). The second indirect effect is the indirect effect of ACEs on suicidal ideation through perceived burdensomeness and was interpreted as significant (*X* → *M*_2_ → *Y*). The third indirect effect is the indirect effect of ACEs on suicidal ideation through CORE, which was interpreted as significant, meaning that CORE-10 score mediated the relationship between ACEs and suicidal ideation (*X* → *M*_3_ → *Y*). The fourth indirect effect is the indirect effect of ACEs on suicidal ideation through thwarted belongingness via CORE-10, which was deemed significant, meaning that thwarted belongingness mediated the relationship between ACEs and suicidal ideation via CORE-10 (*X* → *M*_1_ *M*_3_ → *Y*). The final indirect effect is the indirect effect of ACEs on suicidal ideation through perceived burdensomeness via CORE-10, which was interpreted as significant, therefore perceived burdensomeness mediated the relationship between ACEs and suicidal ideation via CORE-10 (*X* → *M*_2_ *M*_3_ → *Y*).

**Table 3:** Indirect coefficients for the proposed model

Indirect pathways	Effect	95% CI, lower limit	95% CI, upper limit
*X* → *M*_1_ → *Y*^a^	−0.0823	−0.1821	0.0063
*X* → *M*_2_ → *Y*	0.7644	0.4532	1.1331
*X* → *M*_3_ → *Y*	0.0733	0.0132	0.1532
*X* → *M*_1_ *M*_3_ → *Y*	0.0678	0.0246	0.1286
*X* → *M*_2_ *M*_3_ → *Y*	0.0749	0.0301	0.1367

a. Indirect effect is deemed non-significant as 0 falls between the confidence interval.

## Discussion

Research that explores suicide among college students suggests that an interplay of individual, interpersonal and societal factors may increase or decrease suicide risk.^10^ This study aimed to develop a multidimensional model that would explain the association between ACEs and suicidal ideation in college students while considering the mediating factors of thwarted belongingness, perceived burdensomeness and psychological distress. Although previous studies have helped in establishing the relationship between ACEs and suicidal ideation,^19^ this study examined suicidal ideation within the framework of the interpersonal theory of suicide. It aimed to replicate previous findings, as well as fill conceptual gaps in the literature regarding the relationship between being exposed to cumulative ACEs, psychopathology and suicidal ideation.

### Adverse childhood experiences

This study contributes to the small but growing body of research on ACEs in college students by showing that 35.2% of the students reported experiencing 1–3 ACEs and 39.6% reported 4–12 ACEs, out of a list of 12. Thus, the reported prevalence of ACEs in this sample of college students is consistent with that from previous studies in indicating that many young people enter college with prior potentially traumatic experiences that may have an impact on their academic and social life.^9^

The present study also contributes to the existing literature that highlights the relationship between higher cumulative ACE scores and poorer mental health in the form of psychological distress and suicide ideation (e.g.^30^). These findings suggest that ACE scores may be useful as an identifying marker of need for university services so students can get the most out of their time in college. Although students typically underutilise the interventions available to them,^7^ adopting an ACEs perspective for such interventions in the form of integrated promotion strategies and suicide awareness programmes and training could increase their efficiency. However, any attempt to screen for students exposed to potentially traumatic early experiences as part of a trauma-informed approach to college services must be done sensitively, mindful that some students with ACEs may not want, or indeed need, interventions.

### Indirect effects and hypothesis testing

As hypothesised, there was a significant, positive and unique indirect effect of cumulative exposure to ACEs and suicidal ideation through psychological distress in college students. This finding supports previous research indicating that ACEs are associated with future psychopathology.^30^ Borderline personality disorder is one particular form of psychopathology often studied alongside ACEs. Its onset can be attributed to an interaction between biological vulnerabilities and environmental factors such as exposure to trauma in childhood (e.g.^31^). It is suggested that such trauma has relevance in the display of suicide risk among individuals with the condition.^32^

Thwarted belongingness and perceived burdensomeness were included as mediators between ACEs and suicidal ideation via psychological distress. As hypothesised, there was a significant, positive indirect effect of perceived burdensomeness on the relationship between ACEs and suicidal ideation via psychological distress in college students. However, there was a non-significant indirect effect of thwarted belongingness on suicidal ideation, which is consistent with the results of other studies.^29^ However, via psychological distress, the indirect effect of thwarted belongingness was significant in the relationship between ACEs and suicidal ideation. Therefore, these findings suggest that perceived burdensomeness, thwarted belongingness and psychological distress partly explain the association between cumulative exposure to ACEs and suicidal ideation. Although the interpersonal theory of suicide does not propose that thwarted belongingness or perceived burdensomeness differ in their relation with suicidal ideation, it may be of some relevance, from both a theoretical and practical point of view, that perceived burdensomeness is a stronger driver of thoughts of suicide than thwarted belongingness. It is plausible that thwarted belongingness is a less likely experience for college-going individuals than perceived burdensomeness and, thus, counteracting perceived burdensomeness could be given more credence when developing suicide intervention programmes in higher education institutions. Therefore the interpersonal theory of suicide may require some modifications in order to be considered a universal theory of suicide risk.

### Implications for practising clinicians

ACEs have previously been found to be pertinent to a range of adult health outcomes, with high ACEs contributing to the risk of developing chronic health conditions (e.g.^14^). Many studies have indicated that clinicians perceive screening for ACEs to help in developing a deeper clinician–patient relationship and more compassion towards patients and in fostering integrated care, a trusting relationship and better communication.^33,34^ Clinicians have recognised that ACE screening highlights the bridge and connection between mental and physical health, helping to integrate healthcare.^34^ Thus, identifying such adverse experiences may assist clinicians and practitioners in providing more informed, patient-centred, holistic care. Despite some challenges associated with screening, such as patient acceptability, when completed sensitively it can improve patients’ healthcare experiences. In one study, over half of the patients reported that having a conversation about ACE increased trust in their clinician and 75% reported that it helped their clinician know them better.^35^

In the college context or among young adults of college age, the findings of the current study suggest that understanding the association between experiencing ACEs and suicidal ideation and the processes that mediate this association could be beneficial for medical professionals and counsellors in higher education institutions and practitioners working with young adults in psychiatric in- and out-patient units. With regard to perceived burdensomeness, for example, previously shown to be associated with suicidal ideation^5,36^ and also in the present study to be associated with ACEs and psychological distress, it may be worthwhile for clinicians and counsellors involved in individual and group therapy to attune to this aspect of the interpersonal theory of suicide as a means of countering psychological distress and suicidal ideation in individuals with or without trauma histories. Clinicians could employ cognitive restructuring techniques such as cognitive–behavioural therapy with an agenda to modify individuals’ perception of burdensomeness. Further research is also needed to examine the most likely facets of perceived burdensomeness among young adult populations, particularly with regard to who they believe they are a burden to and why.

Social connectedness, a concept related to belongingness, buffers the psychological effects of stressful situations^37^ and is associated with lower levels of suicidal ideation in college students.^36,38^ In one study, researchers found that undergraduate college students identified quality relationships with friends and family as protective factors against suicide.^39^ Acknowledging that loneliness and social isolation can be prevalent on college campuses and pushing efforts to respond to and negate their impact are key avenues of intervention worth pursuing that will benefit the student body at large.

Creating a trauma-informed culture within the college community will foster an awareness of how past adverse experiences can affect students’ present functioning so that appropriate supports can be advertised and provided. Therefore, screening for ACEs, or at least considering their implications, has obvious use in clinical assessment and treatment,^40^ but also in interventions to reduce stress and improve the mental health of the college-going population.

### Limitations

The main limitation of this research is that the sample was largely female and White, restricting the generalisability of findings. Future research could reconfirm the model from this study in more varied population groups. Convenience sampling was used, whereby all individuals who volunteered to participate in the study were allowed to do so. This poses yet another challenge to the generalisability of findings, as students with particular characteristics, such as interest in mental health research or perhaps personal experience of mental illness, may be drawn to the research and bias the results. Lastly, the cross-sectional design of this study does not allow for either causal relationships among key variables to be discerned or the directionality of the associations to be established.

Notwithstanding these limitations, this study supports previous research indicating that individuals who have experienced negative and potentially traumatic life events in childhood are at increased risk for suicidal thoughts and behaviours. It is the first to examine the relationship between ACE exposure and suicidal ideation among college students within the framework of the interpersonal theory of suicide. It establishes key mediating roles for thwarted belongingness, perceived burdensomeness and psychological distress. Future studies should consider replicating our findings using more representative participant sampling and a longitudinal design.

## Data Availability

The data that support the findings of this study are available from the corresponding author on reasonable request.
